# Comparison of diagnostic methods for early interproximal caries detection with near-infrared light transillumination: an in vivo study

**DOI:** 10.1186/s12903-017-0421-2

**Published:** 2017-11-16

**Authors:** Ismail Hakki Baltacioglu, Kaan Orhan

**Affiliations:** 1 Department of Restorative Dentistry, Ankara University, Faculty of Dentistry, Besevler, 06560 Ankara, Turkey; 2 Department of Dentomaxillofacial Radiology, Ankara University, Faculty of Dentistry, Besevler, 06560 Ankara, Turkey

**Keywords:** Nilt, Approximal caries, Photostimulating phosphor, Bitewing radiography

## Abstract

**Background:**

Although numerous studies have used digital intraoral imaging, only a few studies have used photo-optical methods for the diagnosis of caries. Moreover, several limitations exist in terms of observers (experience and specialty) and the caries lesion itself. Hence, the aims of this study were to evaluate the diagnostic capability of near-infrared light transillumination (NILT) and PSP-Bitewing radiographs and to compare the interobserver and intraobserver differences in addition to observers’ experience level to detect early interproximal caries lesions in vivo.

**Methods:**

A total of 52 untreated posterior teeth with and without varying degrees of early interproximal carious lesions were included. Bitewing radiographs using digital phosphor plates (PSP-Bitewing) and NILT were used to clarify the diagnosis. An oral and maxillofacial radiologist and a restorative dentistry consultant evaluated the images twice. A separate appointment for clinical validation and restoration was made. Kappa coefficients were calculated to assess both intraobserver and interobserver agreements for each evaluation method. Scores obtained from PSP-Bitewing and NILT were compared with the clinical validation via receiver operating characteristic (ROC) analysis.

**Results:**

No significant differences were found between PSP-Bitewing radiography and NILT for detecting early interproximal carious lesions with high average Az results. Both intraobserver and interobserver agreement values were relatively higher for NILT evaluation. The Az values increased at second evaluations for both caries detection methods.

**Conclusions:**

NILT examination has an appropriate sensitivity and diagnostic accuracy for detecting early interproximal caries lesions and can be considered as a method of choice for detecting caries without the use of ionizing radiation.

## Background

Early and correct diagnosis of caries affects the choice of treatment to be performed on the patient. Although radiologic evaluation of teeth has a great importance in caries detection, the early caries lesions are difficult to detect, especially using the conventional radiological methodology [1, 2]. In some cases, morphological characteristics could even make it difficult to detect caries. For these reasons, several methods have been proposed for the detection of early caries. Advancements in digital techniques have made a significant contribution to dental radiography [3]. Today, the CCD, CMOS, and PSP systems are routinely used for intraoral imaging. Several previous studies comparing film-based intraoral radiographic images and digital intraoral radiographic images have demonstrated comparable results for caries detection [4, 5]. Some reports discuss the image quality and advantages of CCD systems [6] and PSP systems [7], and some reports indicate the advantages of PSP systems over CCD systems [8, 9]. Although various studies have demonstrated the performances of digital imaging modalities, these imaging modalities still have some limitations such as producing two-dimensional images of three-dimensional objects.

Moreover, according to some reports in the literature, 25%–40% of proximal caries lesions could not be detected by clinical examination without radiologic evaluation. This situation reveals the diagnostic importance of two examinations that have never been apart [10, 11].

Digital radiographs decrease the sensitivity to identify the changes present during the initial stages of lesion progression, and in addition, for interproximal lesions, the X-ray must be focused directly to the approximal region for a proper diagnosis of interproximal caries [12].

The detection and diagnosis of caries using the photo-optical technique was first reported in 1995 [13]. Since then, modifications have improved the imaging quality, and near-infrared light transillumination (NILT) systems were introduced. The NILT method for caries detection is a further development of the digital imaging fiberoptic transillumination method (DiFOTI). The primary difference between these systems is that the DiFOTI system uses visible light, whereas the NILT system uses invisible long-wave light. The major benefit of using longer wavelengths is the decreased scattering, which allows near-infrared light to pass through objects more deeply. None of these systems uses ionizing radiation (X-ray) to visualize the interproximal caries lesions [13–16].

Several studies have used digital intraoral imaging for the diagnosis of caries; however, only a few studies have especially used digital radiography techniques and photo-optical method for caries detection [2, 16–20]. Moreover, there are limited studies in the literature regarding caries detection using both laser fluorescence and NILT [16, 18, 20, 21]. A recent study by Menem et al. [17] tested the diagnostic accuracy of a laser fluorescence device in comparison with bitewing radiographs and concluded that laser fluorescence was significantly better than bitewing radiographs in diagnosing approximal caries. Another recent study by Söchtig et al. [20] also evaluated the bitewing and NILT examination methods. The authors showed that NILT examination can have a similar performance as that of bitewing radiographs to examine both proximal and occlusal surfaces simultaneously.

However, previous studies [16, 17, 20] had some limitations in terms of observers and caries lesions. Almost all studies included only one observer and did not take into account the experience level and specialty for diagnosing caries lesions. Moreover, the available literature evaluated the progressed caries lesions, and to the best of our knowledge, only a few studies have been conducted for the detection of early caries lesions comparing NILT and bitewing radiographs with clinical validation [16, 20].

Hence, the aims of this study were to evaluate the diagnostic capability of NILT and PSP-Bitewing radiographs and to compare the interobserver and intraobserver differences in addition to observers’ experience level to detect early interproximal lesions in vivo.

## Methods

Using retrospective data of the literature, a power analysis (Power and Precision software, Biostat, Englewood, NJ, USA) was conducted that indicated that the detection of differences between two caries detection modalities could be obtained with at least 50 teeth at a power of 0.8 (alpha = 0.05). Thus, this study was initially conducted on 70 teeth of 35 patients. This study was approved by the Ethical Committee of Dentistry Faculty (Ethical Clearance Number 10/7, 2016) and followed the principles of the Declaration of Helsinki, including all amendments and revisions. Collected data were only accessible to the researchers.

The patients were selected from the outpatient clinic of the Faculty of Dentistry, Ankara University. The patient-related inclusion criteria were fully erupted permanent dentition and a minimum age of 18 years. Teeth with restorations and large cavitations and caries were not included in the study. As a standard care, intra/extra oral examination with bitewing radiographies are performed to all patients who admitted to outpatient clinic. All patients provided written informed consent before undergoing any radiographic, intraoral, or extraoral examinations. The bite-wing radiographs was taken using Phosphor Plate radiography (PSP-Bitewing) (Digore Optime, Soredex, Helsinki, Finland at 60-kV tube potential, 7 mA, and with 0.064 s exposure time with using a PSP sensor holder (XPP-DS Digital Sensor Holders for Sirona, Dentsply, IL, USA).

The teeth that were suspected for early interproximal dentin caries lesions from bitewing radiographs with no observable cavity lesions during visual examination in the posterior teeth were selected for inclusion in the study. Further written approval was taken from the patients to be part of the study.

An oral and maxillofacial radiologist (KO) and a restorative dentistry consultant (IHB) evaluated the images twice. The radiologist consultant has 16 years of experience in diagnosing and evaluation of caries lesions using all radiographic modalities. The restorative dentistry consultant has 8 years of experience in diagnosing caries lesions both clinically and radiographically. The observers were selected from different specialties dealing with caries detection as a daily routine activity in their clinical environments. Hence, it was considered worthwhile to compare between these two specialists for early caries detection in the daily clinical practice.

The observers were blinded with regard to the clinical status of the patients. Before starting the study, a calibration was made between the observers. They received a set of guidelines and classification criteria for caries. The observers evaluated the images according to caries detection modalities.

The following scale was applied for the detection of caries: (1) definitely caries, (2) probably caries, (3) uncertain, (4) probably no caries, and (5) definitely no caries.

**Fig. 1:**
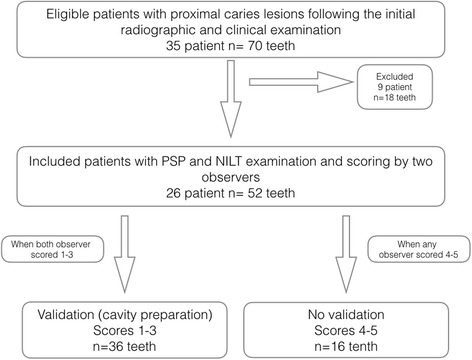
The flow chart for methodology

If a prediagnosis was made between scales 1–3, all the treatment methods were discussed with the patient. If both observers had reached a consensus for the operative procedure, the patients were asked whether they could participate in this study. An informed consent document was obtained from those who wished to participate, and a separate appointment was arranged for the validation and restorative treatment. A flow chart showing the methodology of the study is depicted in Fig. 1.

### Study population

Nine patients who did not match the participation criteria (those who did not provide the informed consent and those who missed the appointments) were excluded from the study. A total of 26 patients (9 males with a mean age of 28.1 years and 17 females with a mean age of 32.4 years) with early interproximal caries lesions were included in this study. From these patients, 52 untreated posterior teeth without any observable cavitations that had various degrees of caries lesions were included in the study.

### Clinical examination

During this appointment, the suspected area was reevaluated clinically and radiographically prior to the validation phase. The validation phase was done only for the suspected lesions with a scale of 1–3, which was diagnosed by two observers. Non-cavitated approximal lesions and equivocal radiolucencies nearby the dentoenamel junction were removed from this study to prevent overtreatment. The decision for cavity preparation (validation phase) was made in combination with clinical and radiographic evaluation of both observers.

### NILT examination

**Fig. 2:**
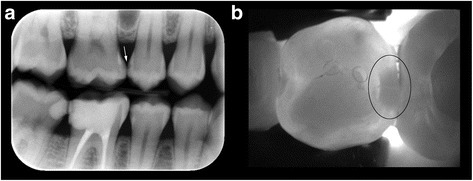
**a** PSP-Bitewing radiograph showing proximal caries lesions in both the second premolar (scale 1) and first molar (scale 3) (arrow), (**b**) NILT image showing the carious lesion only for the second premolar (scale 1) and without any caries lesion (scale 5) for the first molar teeth

Images were obtained and recorded using the KID software (KaVo Integrated Desktop/version 2.4.1.6374, KaVo, Biberach, Germany) after air-drying, using the NILT camera, at different angles from the approximal regions of related teeth. Following the clinical and radiological phase, the NILT images were analyzed by the two observers within 2 days from the other diagnostic findings including PSP-Bitewings (Fig. 2).

The same scoring system was used again as follows: (1) definitely caries, (2) probably caries, (3) uncertain, (4) probably no caries, and (5) definitely no caries. In addition, the NILT images were analyzed by another restorative dentist to achieve a consensus diagnosis. If the observers gave different scores, these scores were noted to compare with the diagnosis after the validation phase; in contrast, if any observer gave 4 or 5 scores, no validation was performed on the relevant tooth. The validation process was started after reaching a consensus among the observers.

### Clinical validation

The validation process included opening the interproximal dentin lesion using a round diamond burr to confirm the presence of the lesion. Following this evaluation, the caries process was completely excavated and a final cavity was obtained. The prepared cavity was restored with a composite restoration (Clearfil Majesty Esthetic, Kuraray, Tokyo, Japan). The restorative dentist (IHB) carried out the clinical validation and the restorative treatment.

### Statistical analysis

Kappa coefficients were calculated to determine both interobserver and intraobserver agreements for each examination method (PSP-Bitewing and NILT). The kappa values were performed using the protocols of Landis and Koch adapted by Altman [22] as follows:

Poor: ≤0.20.

Fair: 0.21–0.40.

Moderate: 0.41–0.60.

Good: 0.61–0.80.

Very good: 0.81–1.00.

Receiver operating characteristic (ROC) analysis was used to evaluate the observers’ performance for distinguishing the teeth with or without approximal caries. PSP-Bitewing and NILT scores were compared with the clinical validation. Since lower values are obtained from the existing scoring system and higher values are required for ROC analysis, the areas between 0.5 (no caries [scores 4–5]) and 1 (caries/uncertain [score 1–2-3]) were used for the analysis. Cutoff values were obtained by examining any value of the ROC curve with regard to its sensitivity and specificity, and subsequently, the predictive values were calculated. The A_Z_ values were calculated by GraphPad Prism 6 for Mac OS X software (GraphPad Software, Inc. La Jolla, CA, USA), and the A_Z_ values for each evaluation method, observer, and readings were compared using z-tests, which was described by McClish et al. [23]; *p* values <0.05 were interpreted as statistically significant.

## Results

A total of 36 teeth were sent for the validation process for detecting early caries lesions (scores 1–3). The remaining teeth were diagnosed with scores 4–5 after evaluation by all the methods and the consensus of all the observers. Of the 36 teeth from the total 52, which were evaluated through examinations, methods were identified as early caries lesions after clinical validation.

Intraobserver kappa coefficients for the evaluation type are shown in Table 1. Good intraobserver agreement was achieved between the PSP-Bitewing and NILT evaluation methods. Intraobserver kappa coefficients ranged from 0.554 to 0.646 for PSP-Bitewing, while those for NILT ranged from 0.792 to 0.884, suggesting noticeably good intraobserver agreement. Interobserver kappa coefficients for both the first and second readings according to the evaluation methods are shown in Table 2. NILT showed higher interobserver agreement when compared with PSP-Bitewing. Good interobserver agreement was obtained for the first and second readings for both methods.

**Table 1:** Intra-observer agreement calculated for each observer according to evaluation methods

	Observer 1		Observer 2	
	Kappa	Standard Error	Kappa	Standard Error
PSP-Bitewing	0.646	0.017	0.554	0.022
NILT	0.792	0.044	0.884	0.058

**Table 2:** Inter-observer kappa coefficients among the observers for the first and second readings

	First Reading Observer1-Observer 2		Second Reading Observer1-Observer2	
	Kappa	Standard Error	Kappa	Standard Error
PSP-Bitewing	0.618	0.018	0.673	0.017
NILT	0.788	0.042	0.746	0.038

Table 3 shows the areas under the ROC curves (Az values) for both the observers, readings, and evaluation methods. Although high Az values were obtained for both methods, higher values were obtained for the NILT method. Table 4 shows the comparisons between the modalities with Az values and the significance between them. The highest Az values were obtained from the second reading of the second observer for the NILT method. The Az values increased for both evaluation methods for the second readings.

**Table 3:** AZ values, standard errors, and significance levels for all observers and their readings

	Observer 1						Observer 2					
	First Reading			Second Reading			First Reading			Second Reading		
PSP-Bitewing	0.630	0.020	<0.0001	0.682	0.0142	<0.0001	0.786	0.0122	<0.0001	0.6730	0.0119	<0.0001
NILT	0.785	0.034	<0.0001	0.803	0.0156	<0.0001	0.832	0.0187	<0.0001	0.822	0.0162	<0.0001

**Table 4:** Comparisons between modalities using z-tests with a significance level of 0.05

*p* values				
	Observer1-1st reading	Observer1-2st reading	Observer2-1st reading	Observer2-2st reading
PSP-Bitewing	0.0180	0.2520	0.0367	0.3082
NILT	0.0292	0.6251	0.0388	0.3047

## Discussion

Radiologic evaluation of teeth has a great importance in addition to clinical examination, especially for the detection of early proximal caries, which could be difficult with intraoral examination. Intraoral imaging modalities have some limitations; they provide two-dimensional images of three-dimensional structures [24, 25]. The evaluation of dental structures with conventional two-dimensional images when reading these radiographs for early carious detection, a third dimension is important to determine the lesions. The evaluation of dental structures using conventional two-dimensional images when interpreting these radiographs, especially early carious lesions, a third dimension is crucial to identify the lesions [26, 27].

Conventional film-based imaging techniques have been used over several decades, but in conjunction with the technological development, they have left their importance to digital imaging techniques. Digital sensors provide significant benefits such as low radiation dose, easy storage, and possibility of managing images [24, 26, 28]. Several recent studies comparing film-based intraoral radiographic images and digital intraoral radiographic images have demonstrated comparable results for caries detection. Some reports discuss the image quality and the advantages of CCD systems and PSP systems, and some reports indicate the advantages of PSP systems over CCD systems [4, 6–9].

Although bitewing radiographs are accepted as the gold standard for the detection of proximal caries, the issue of ionizing radiation should still be considered for every single examination. It has been clearly shown that the effective dose was approximately 13 mSv from a panoramic radiograph, 1–3 mSv from a cephalometric radiograph, 1–8 mSv from a periapical radiograph, and 8 mSv from an occlusal radiograph [29, 30] According to the principle of “as low as reasonably achievable,” radiographic examinations must be performed only when they are needed and evidence-based selection criteria should be considered. Moreover, although several studies [17, 31, 32] have been conducted regarding the correlation between bitewing radiolucency and cavitation status in approximal caries, no study has presented strong evidence for concluding a clinical threshold at which the restoration of approximal caries can be recommended.

Several nonionizing techniques such as fiberoptic transillumination (FOTI), quantitative light-induced fluorescence (QLF), and electrical conductance (EC) have been tested for their diagnostic accuracy in detecting approximal caries [33–35]. Recently, another new technology, the near-infrared laser technology, has become an alternative to fluorescence methods; however, studies evaluating and comparing these technologies are still limited. The comparison between bitewing radiographs as the gold standard and NILT is also limited since each of these methods uses different principles because of the changes in the tooth tissue due to the presence or absence of carious lesions. Therefore, a comparison of these methods is crucial for assessing the reliability of carious detection.

The present study compared the diagnostic accuracy of PSP and NILT examination methods for the detection of early caries lesions without any clinical visibility of the proximal carious lesions. Both intraobserver and interobserver agreement values for NILT were relatively better than those with PSP intraoral radiographies. The highest kappa values were obtained with NILT examinations. Bussaneli et al. [19] compared the feasibility of using NILT in the diagnosis of incipient carious lesions with that of digital radiographic examination. They found that the NILT method had higher reproducibility than the radiographic examination. A similar result was also reported by Maia et al. [36]. However, in both studies, NILT presented lower interobserver reliability. The present study is consistent with the previous studies. NILT examinations showed better performance than that of PSP-Bitewing radiographs, although with a moderate interobserver reliability. The results showed that the second readings were better than the first readings, with higher kappa values. This may be explained by the fact that both observers obtained more experience in evaluating the NILT images and improved in the second readings.

Söchtig et al. [20] also evaluated the bitewing and NILT examination methods. They showed that the NILT examination can have a similar performance compared to that with bitewing radiographs, which can be repeated as often as necessary and provides the opportunity to examine both proximal and occlusal surfaces simultaneously. In that paper, the authors indicated two limitations; one is the lack of a control group, and the other is the clinical validation. Our study represented the clinical validation of the carious lesions. Of the total 52 teeth, 36 teeth were initially diagnosed using all the evaluation methods and by the consensus of the examiners and were clinically validated as having carious lesions. Kühnisch et al. [16] also conducted an in vivo study as the current study. Due to ethical rules, no validation process was carried out for negative results, sound regions, and initial enamel caries. Because of missing negative test results, the specificity values could not be calculated in the study by Kühnisch et al. [16].

### Limitation of the study

There are several limitations in this study. Because of not clinically validating the teeth that were scored as (4) probably no caries and (5) definitely no caries, there may still be missing caries lesions in these patients. However, because of ethical reasons, one cannot clinically validate these lesions because of unnecessary interventional possibility to the patients. Another limitation is that only two observers were included in the study, a consultant of maxillofacial radiology and a restorative dentistry consultant. More number of consultants or even a general dentist with a difference experience level should have been included in this study. In addition, since various radiographic and several NILT images were used in this study by the observers, a nonbiased investigation of the intraobserver and interobserver reproducibility was not possible.

Additional studies should be conducted with inclusion of different specialists and general dentists with different experience levels for diagnosing the caries lesions. A nonbiased observer reliability methodology should be set up in future studies. Moreover, to test the scores 4 and 5, pretreatment orthodontic extractions can be used for clinical validation studies using NILT.

## Conclusion

Based on the study findings, it can be stated that NILT can be useful for confirming the absence of proximal caries when bitewing radiography is questionable. The study results also revealed the good performance of NILT in the detection of early carious lesions, with values of accuracy and area under the ROC curve similar to those of the bitewing method. The NILT method can be recommended as a valid alternative for the diagnosis of early caries lesions on the proximal surface of permanent teeth without radiation exposure.

## Abbreviations

CCD: Charge couple device
CMOS: Complementary metal oxide
DiFOTI: Digital imaging fiberoptic transillumination method
EC: Electrical conductance
FOTI: Fiberoptic transillumination
NILT: Near-infrared light transillumination
PSP: Photostimulable phosphor plates
QLF: Quantitative light-induced fluorescence
ROC: Receiver operating characteristic
